# Cost of Nosocomial Outbreak Caused by NDM-1–Containing *Klebsiella pneumoniae* in the Netherlands, October 2015–January 2016

**DOI:** 10.3201/eid2309.161710

**Published:** 2017-09

**Authors:** Madelief Mollers, Suzanne P. Lutgens, Annelot F. Schoffelen, Peter M. Schneeberger, Anita W.M. Suijkerbuijk

**Affiliations:** European Centre for Disease Prevention and Control, Stockholm, Sweden (M. Mollers);; National Institute for Public Health and the Environment, Bilthoven, the Netherlands (M. Mollers, A.F. Schoffelen, A.W.M. Suijkerbuijk);; Jeroen Bosch Hospital, ’s-Hertogenbosch, the Netherlands (S.P. Lutgens, P.M. Schneeberger)

**Keywords:** nosocomial, HRMO, *Klebsiella pneumoniae*, CPE, *Enterobacteriaceae*, economic, costs, outbreak, the Netherlands, NDM-1, antimicrobial resistance, bacteria, extended-spectrum β-lactamase, ESBL

## Abstract

During October–December 2015, 29 patients in a hospital in the Netherlands acquired nosocomial infection with a multidrug-resistant, New Delhi-metallo-β-lactamase–positive *Klebsiella pneumoniae* strain. Extensive infection control measures were needed to stop this outbreak. The estimated economic impact of the outbreak was $804,263; highest costs were associated with hospital bed closures.

In 2008, New Delhi-metallo-β-lactamase (NDM), an enzyme that confers bacteria with resistance to a range of antimicrobial drugs, was detected for the first time in a patient from Sweden during a trip to India (*1*). Subsequently, NDM-producing isolates rapidly spread and have been found dispersed throughout the world. However, in western and northern Europe, identification of patients with NDM-producing *Enterobacteriaceae* is uncommon (*2*). Infections with multidrug-resistant, gram-negative bacteria are a concern worldwide, given restricted treatment options and excess costs of care (*3*,*4*). 

During October 1–December 30, 2015, an outbreak of *Klebsiella pneumoniae* containing an NDM-1 plasmid affected 29 patients residing in Jeroen Bosch Hospital (’s-Hertogenbosch, the Netherlands), a 683-bed tertiary teaching hospital. This hospital outbreak started in a surgical ward. On November 23, 2015, NDM-producing extended-spectrum β-lactamase (ESBL)–positive *K. pneumoniae* bacteria were cultured and isolated from surgical drain fluid. At the time of identification, the patient was already discharged. Shortly thereafter, screening cultures of long-term admitted surgical patients revealed 2 additional patients with NDM-producing *K. pneumoniae*. Contact tracing and weekly screening rounds of all in-hospital patients were performed, identifying additional NDM carriers. Weekly screening rounds revealed 7 wards with uncontrolled NDM transmission (i.e., >2 NDM carriers). On the basis of an epidemiologic curve of the NDM carriers detected, all patients admitted to 1 of these wards beginning October 1 were defined as at risk of carrying NDM. Because the policy that was chosen was search and destroy (detect patients as quickly as possible and isolate them to protect the others), all patients residing at high-risk wards were tested.

Six months after the start of the outbreak, 2,964 patients had been flagged as at-risk patients; >95% of these patients had been screened, and a total of 29 NDM carriers were identified. No risk factors, such as recent travel abroad or a common source of transmission, were identified among the cases of this outbreak. In 2016, weekly screening rounds were continued in wards with at-risk populations to confirm the outbreak was successfully controlled.

Apart from the physical burden to patients and hospitals caused by multidrug-resistant microorganisms, nosocomial outbreaks also entail an economic burden. Estimates of the cost of outbreaks of multidrug-resistant bacteria in healthcare institutions are scarce. Insight on outbreak costs can help to justify the necessary investments in infection prevention and control measures, facilitating the decision-making process on prevention and control policy. In this study, we assessed the total costs of this outbreak on the basis of interviews and data from the affected hospital.

## The Study

The outbreak occurred in a hospital with 683 registered beds, including a separate rehabilitation center. We assessed outbreak-related costs by using an activity-based costing model and performed interviews with staff working in the hospital to gather additional information about outbreak control activities performed and costs (Technical Appendix). We calculated hospital costs from October 1, 2015, the beginning of the outbreak, through January 31, 2016, one month after the end of the outbreak, when the greater part of costs had been made. We divided outbreak costs into diagnostics costs, ward-related costs, and other outbreak-related control measure costs. All costs are expressed as 2015 US dollars and Euros. Euros were converted to US dollars by using the data on the purchasing power parity of the Organization for Economic Co-operation and Development (https://data.oecd.org/conversion/purchasing-power-parities-ppp.htm): €1 = US $1.23.

The laboratory of the hospital performed diagnostic tests (bacteria cultures and PCR tests) and antimicrobial drug susceptibility testing for patients. All PCR tests were performed in batches. Items that were included in the determination of the costs of diagnostics were testing materials, procedures, and laboratory personnel. Personnel time of the microbiologists was valued by multiplying the time spent on laboratory and outbreak management activities, as quoted during the interviews, by unit costs per hour, taken from Dutch guidelines for economic evaluations (*5*).

We retrieved loss of revenues caused by closed beds after the outbreak from the hospital database and list prices online (*5*) and adjusted this number for the occupancy rate of the hospital, which was 85% on average. The extra expenses for personal protective equipment (disposable aprons, gloves, and masks) and cleaning the wards affected by the outbreak were gathered by the department of technical and facility services.

We also included costs associated with the additional time spent by healthcare workers on patient isolation. Following Wassenberg et al., we assumed 30 min/d for nurses and 10 min/d for physicians as the time required for adhering to control measures (*6*). The infection prevention expert provided the number of staff meetings in which outbreak interventions were discussed and the number of employees participating in these meetings. Both the executive manager and the communication manager provided data on the amount of time associated with outbreak response activities. Finally, other costs included costs for sending test kits to persons who had been hospitalized in the outbreak period.

We estimated total outbreak costs at $804,263 or €653,801 (Table), corresponding to a cost of $27,700 per patient. The loss of revenues due to of closure of beds contributed the most to the total costs. Other cost drivers were diagnostic tests and personnel time spent by laboratory employees and infection prevention experts.

**Table:** Total outbreak costs stratified by type of cost, Jeroen Bosch hospital, the Netherlands, Oct 2015–Jan 2016*

Type of cost	Explanation	Total cost, US $	Total cost, €
Diagnostics			
Other laboratory personnel	Estimated 2,517 h†	93,789	76,251
Microbiological tests	Material costs to perform cultures in batches	60,070	48,837
Microbiologists	Estimated 376 h†	46,017	37,412
Molecular diagnostics	Material costs to perform PCRs in batches	24,523	19,937
Subtotal diagnostics		224,399	182,437
Ward-related costs			
No. blocked beds	582 beds, occupancy rate 0.85 at $550/d or €447/d (*5*)	272,085	221,131
Personal protective equipment	Expenditures for extra disposable aprons, gloves, and masks	55,121	44,814
Cleaning wards	Purchase of 2 fogging devices and personnel time for extra cleaning	46,881	38,115
Subtotal ward-related costs		374,087	304,060
Other outbreak control costs			
Infection prevention experts	Estimated 2,336 h for internal advice and guidance†	105,356	85,655
Patients in isolation	280 patients, averaged at 5.2 d of hospitalization, at $31.40/d or €25.53/d (*6*)	45,718	37,172
Staff meetings	23 staff meetings with on average 21 participants × 0.75 h × $1,525/h†	26,306	21,390
Communication	320 h for internal and patient-related communication spent by several communication employees†	17,696	14,387
Costs for mailings		10,701	8,700
Subtotal outbreak control costs		205,777	167,304
Total costs		804,263	653,801

*Resource use related to this outbreak was provided by the hospital. †Labor costs/h were determined by using the Dutch manual for economic evaluations (*5*).

## Conclusions

The NDM-1 outbreak at Jeroen Bosch Hospital in the Netherlands in 2015 was associated with substantial costs incurred by the hospital, estimated at $804,263 or €653,801, which was 12% of the total budget allocated that year for medical microbiology and infection prevention, and $27,700 per patient. Blocked beds had the highest effect on the total costs, followed by staff time targeted at infection prevention activities.

A few studies have evaluated outbreak costs in hospitals; however, none of these were targeted at NDM outbreaks. Compared with other studies on the costs of hospital outbreaks with other pathogens, such as *Acinetobacter baumanni* (*7*,*8*), norovirus (*9*), ESBL-producing *K. pneumoniae* (*9*), and *Enterococcus faecium* (*9*), our estimates are higher. One major factor explaining this difference was the testing of a relatively high number of patients; the closure of beds was the main cost driver in all applicable studies.

Despite being substantial, the cost we calculated for the outbreak is an underestimate. At least 9 NDM-1–positive patients and 28 other patients were discharged to a long-term care facility resulting in additional infection control measures and costs that were not taken into account for this report. In addition, a medical doctor, infection prevention expert, and infectious diseases nurse of the Municipal Health Service spent 95 h, 65 h, and 30 h, respectively, on the outbreak, accounting for $9,551 additional costs. Furthermore, phylogenetic molecular methods were performed at the National Institute of Public Health to confirm the outbreak. Finally, we only calculated the outbreak costs through January 31, 2016, but additional costs probably were incurred after this date.

As shown in this study, the expansion of multidrug-resistant, gram-negative bacteria is of great concern; these bacteria both threaten patient safety and increase healthcare costs. The intensive outbreak control measures of the hospital were costly and inconvenient for patients and staff. In countries where NDM-1–positive *K. pneumoniae* is not endemic, early detection of colonized patients and adequate infection prevention control strategies will be key factors in minimizing the spread of multidrug-resistant bacteria.

Technical Appendix Description of interviews conducted with hospital personnel.
