# Supplementary material for: Cost of Nosocomial Outbreak Caused by NDM-1–Containing Klebsiella pneumoniae in the Netherlands, October 2015–January 2016
Source: Emerg Infect Dis. 2017 Sep;23(9):1574–6. doi: 10.3201/eid2309.161710 (PMC5572861; doi:10.3201/eid2309.161710)
Supplement: Technical Appendix — Description of interviews conducted with hospital personnel. [file 16-1710-Techapp-s1.pdf]

Article DOI: https://doi.org/10.3201/eid2309.161710

Cost of Nosocomial Outbreak Caused by NDM-1–Containing *Klebsiella pneumoniae* in the Netherlands, October 2015–January 2016

Technical Appendix

Additional Methods of Data Collection

At first, we contacted the microbiologist of the hospital and asked for the right persons involved with the outbreak who could give us additional information about outbreak control activities performed and costs. Second, we emailed the relevant employees for their willingness to participate in this cost evaluation and invited them for an interview. In preparation of the interview, we sent them the questionnaire and asked for available data on costs.

Face-to-face interviews were held with the general business manager of the hospital, a medical microbiologist, the manager of technical and facility services, a person accountable for planning of hospitalizations, the head nurse of the hospital ward mostly affected by the outbreak, an infection prevention expert, and the executive manager responsible for the laboratory personnel and infection prevention. In addition, the communication manager and the Municipal Health Service physician were interviewed by phone and through email.

The interviews were conducted in February and March 2016. At that time, all outbreak activities were over.
